# Performance Characteristics of *Ankistrodesmus falcatus* in Different Culture Media and Concentration

**DOI:** 10.3390/plants10040755

**Published:** 2021-04-13

**Authors:** Victor Tosin Okomoda, Ali Kerdasi Abdulrahman, Helena Khatoon, Sukumaran Mithun, Abraham Sunday Oladimeji, Ambok Bolong Abol-Munafi, Korede Isaiah Alabi, Cosmas Chidiebere Alamanjo, Hassan Anuar

**Affiliations:** 1 Department of Fisheries and Aquaculture, College of Forestry and Fisheries, University of Agriculture, P.M.B. 2373 Makurdi, Nigeria; 2 Higher Institution Centre of Excellence (HICoE), Institute of Tropical Aquaculture and Fisheries Research (AQUATROP), Universiti Malaysia Terengganu, Kuala Nerus 21030, Terengganu, Malaysia; alikerdasi@yahoo.com; 3 Department of Aquaculture, Faculty of Fisheries, Chittagong Veterinary and Animal Sciences University, Khulshi, Chittagong 4225, Bangladesh; helena@cvasu.ac.bd; 4 Department of Aquatic Biology and Fisheries, University of Kerala, Kariavattom, Thiruvananthapuram 695581, Kerala, India; mithunsugun@gmail.com; 5 Agricultural Department, National Biotechnology Development Agency (NABDA), Abuja, P.M.B. 5118 Abuja, Nigeria; sunnybleek2013@gmail.com; 6 Faculty of Food Science and Fisheries, Universiti Malaysia Terengganu, Kuala Nerus 21030, Terengganu, Malaysia; 7 Department of Agricultural Extension and Management, Federal College of Forestry, P.M.B. 2019 Jos, Nigeria; korrexy4ever@yahoo.com; 8 Department of Agricultural Technology, Federal College of Forestry, P.M.B. 2019 Jos, Nigeria; cosmas.alamanjo@fcfjos.edu.ng

**Keywords:** microalgae, specific growth rate, biochemical composition

## Abstract

This study determined the effect of growth media and culture concentration on the growth, proximate, and microelement composition of *Ankistrodesmus falcatus*. The culture of *A. falcatus* was done using three media, namely Modified COMBO Medium (COMBO), Bold’s Basal Medium (BBM), and Bristol, at two concentrations (50% and 100%). The results obtained show that the cell density (>3.5 × 10^7^ cells/mL), optical density (>0.24), and specific growth rate (>0.429%/day) were significantly higher (*p* ≤ 0.05) in BBM and COMBO than in Bristol (<3.1 × 10^7^ cells/mL; <0.23; <0.416%/day, respectively) at both concentrations. However, biomass was higher in BBM (>2.20 g/L) than in COMBO (1.87–2.13 g/L), while Bristol had the lowest value observed (1.70–1.73 g/L). Biochemical and microelement composition showed variations between media and at the different concentrations, with higher values observed in BBM and COMBO. Based on the growth parameters and nutritional composition, it was concluded that BBM and COMBO were better media for the propagation of *A. falcatus* growth than Bristol. The study also demonstrated that the microalgae can be cultured using half of the media’s concentration to lower production costs.

## 1. Introduction

Microalgae, like other photosynthetic organisms, convert solar energy into stored chemical energy [1]. They are diverse groups of unicellular and multi-cellular microscopic heterotrophs/autotrophs which constitute the primary producers of an aquatic ecosystem [2,3]. Over forty thousand species of microalgae have been identified, many of which possess high nutritional characteristics [4,5,6]. The microscopic size gives them a high surface area to volume ratio which enhances the rapid uptake of nutrients and faster growth of their cell [7]. Consequently, they have high photosynthetic efficiency, rapid growth, higher lipid content, high carbon dioxide mitigation efficiency [8,9,10,11,12], and the capacity to grow in saline waters [13,14,15].

The current interest in microalgae mass production and its application in food, pharmaceuticals, cosmetics, aquaculture, and horticulture sectors is predicated on its possession of bioactive chemical constituents [16]. They are natural producers of protein, lipid, carotenoids, and fatty acids, which are essential in human and animal nutrition as well as medicines [17]. The high oil yield of many microalgae species has been exploited in biofuel production [18,19,20]. The advantages of biofuel from microalgae over other conventional biofuel sources are based on its non-seasonality, ease of large-scale biomass production, biodegradability, renewability, non-toxic nature, and less competitive use [21,22,23,24]. Microalgae have also found a place in the aquaculture sectors as they are used to enhance the flesh and skin color of the cultured fish [25]. The possession of high levels of sterols, essential fatty acids, and minerals also make several microalgae species excellent food for larvae of many fin fish and shellfish [17,26].

Similarly, antibacterial effects against aquaculture pathogens have been reported with some phytoplankton species [27,28]. The freshwater phytoplankton *Ankistrodesmus* sp., for instance, has been demonstrated to inhibit the growth of *Streptococcus agalactiae* in the study by Sharifah et al. [29]. The microalgae species *Ankistrodesmus falcatus*, in particular, has been extensively studied for its potential in the production of biodiesel due to its high lipid content and biomass productivity [13,30]. These studies demonstrate the potential of the microalgae *Ankistrodesmus* sp. as an alternative to chemical antibiotics and as an important source of green energy.

The various advantages and potential uses of microalgae have inspired research into the mass production of different marine and freshwater algae species. More so, the biochemical composition of microalgae varies based on factors such as species differences, culture conditions, as well as the composition of culture media [17]. It is well known that different autotrophic media can significantly impact the growth performance characteristics of various microalgae species [31], to this effect, different culture media have been developed for the cultivation of microalgae. Therefore, identifying better media alternatives and growth conditions for culture are steps towards achieving mass production of any microalgae species. In this study, we reported the effect of culture media and concentration on the growth and biochemical parameters of *A. falcatus.*

## 2. Materials and Methods

### 2.1. Growth Media for A. falcatus Production

**Figure 1:**
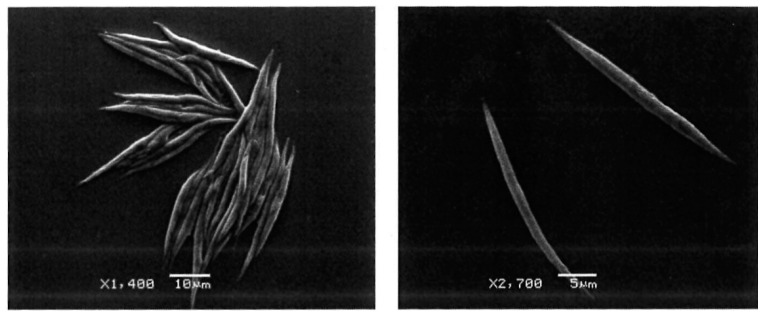
Pure culture of *Ankistrodesmus falcatus* viewed under a scanning electron microscope.

Pure culture of *A. falcatus* (Figure 1) was obtained from the microalgae laboratory at the Institute of Tropical Aquaculture and Fisheries of the Universiti Malaysia Terengganu. Three growth media were used at different concentrations (50% and 100%) in this study. They are, namely, Modified COMBO Medium (COMBO), Bold’s Basal Medium (BBM), and Bristol (Table 1). Preparation of the growth medium was done in 1 L conical flasks with the addition of 21 mL aliquot of the microalgae, before making up the total volume to 700 mL in each of the three replicates used for the different treatments. The conical flasks were then covered using a clean sterilized sponge to let out NH_3_ and reduce/prevent contamination. Continuous aeration was provided to all the experimental conical flasks using a blower sonic p-85 (Air-pump^©^) and filtered using 0.20 µm. The temperature was fixed at 21 ± 1°C throughout the study. Large fluorescent light tubes with a power of 1700 Lux (34 µmol m^-2^ s^−1^) were used to provide adequate light intensity for the culture. A light meter (DT-1309) was used to measure and ensure equal lighting for all replicates of the treatments. Three times daily, each conical flask in the setup is manually shaken to avoid algal residual accumulation at the bottom, which could cause algae mortality. All the treatment groups were prepared and incubated under the same environmental condition for nine days.

### 2.2. Growth Performance Evaluation

Analysis of growth potential of the microalgae in the various treatments was in terms of cell count (cells/mL), optical density (680 nm), biomass (g/L), and specific growth rate (%/day). The sample cell was estimated using a hemacytometer (Hawksley AC1000, Lancing, UK) according to the method by Lavens and Sorgeloos [35]. Optical density was determined using a spectrophotometer (Shimadzu UV-1601, Tokyo, Japan) with a wavelength of 680 nm [36]. The biomass was also obtained through the method specified by Lavens and Sorgeloos [35]. These parameters were obtained from the day the experiment was set up (day 0) until the end of the study at the specific time set for daily harvesting. The specific growth rate (SGR) of the microalgae was calculated at the end of the growth study using the formula given by Banerjee et al. [37], as stated below Equation (1):

(1) $$\mathrm{Specific}\;\mathrm{growth}\;\mathrm{rate}\;(\%/\mathrm{day})\;=\;\frac{log_{e}\left(X_{2}\right)-log_{e}\left(X_{1}\right)}{t_{2}-t_{1}}$$

where:

*X_1_* = biomass concentration at the beginning of the selected time interval;
*X_2_* = biomass concentration at the end of the selected time interval;
*t_2_* − *t_1_* = the selected time for the determination of biomass of microalgae species.

### 2.3. Proximate Composition and Microelement Analysis

For the proximate and microelement analysis, three samples from each replicate of each treatment were used for these analyses. The method of Lowry et al. [38] was employed for the determination of protein in this study. For lipid analysis (%), the sulfuric acid charring method proposed by Marsh and Weinstein [39] and the carbonization method using tripalmitin as the standard after extracting lipids proposed by Bligh and Dyer [40] were used. Carbohydrate analysis (%) was done according to Dubois et al. [41], while the elemental composition (mg/L) of *A. falcatus* was estimated using Inductively Coupled Plasma Mass Spectroscopy (ICP-MS) (Perkin Elmer, Waltham, MA, USA) according to Arslan et al. [42].

### 2.4. Statistical Analysis

All statistical analyses regarding the growth parameters, proximate composition, and microelement composition was performed using the statistical data analysis software Minitab 14. Descriptive statistics were done for the various treatments; thereafter, a test for normality and homogeneity of variance was done. Upon confirmation of conformity, the collected data were analyzed using a two-way analysis of variance (ANOVA). Significant differences amongst treatments were determined and mean separated using Fishers Least Significant Difference at 0.05 levels. The proximate composition parameters, however, were analyzed using the non-parametric Friedman test. The paired Wilcoxon test was then used to identify significant differences within the means.

## 3. Results and Discussion

### 3.1. Biomass and Growth Characteristics of A. falcatus in Different Culture Media

The selection of a suitable growth medium is an important factor to consider when trying to enrich any microalgae [43]. The growth of *A. falcatus* in terms of cell density (Table 2) was higher in BBM and COMBO (3.5 × 10^7^ to 3.9 × 10^7^ cells/mL) compared to Bristol (3.0 × 10^7^ to 3.1 × 10^7^ cells/mL). George et al. [44] had earlier reported that *A. falcatus* grown in different media and under different photoperiod regimes showed better growth in BG-11 (1.62 × 10^7^ cells/mL) compared to BBM (3.04 × 10^5^ cells/mL). Also, using the growth media BG-11, Talukdar et al. [45] reported 1.5 × 10^6^ cells/mL for *A. falcatus* after 10 days of culture. The same trend of performance in cell density was observed in the other growth indices measured in our study and was higher than the reports of Talukdar et al. [45] for *A. falcatus* in BG-11 medium (0.2 d^−1^). The differences in the growth of the different media are likely due to the apparent differences in the media’s composition. Khatoon et al. [43] had stated earlier that the preference of a medium by microalgae mainly depends on the chemical composition of the medium, among other factors. Bristol was lacking in most trace elements essential for the growth of a wide range of microalgae; therefore, this could be the reason for the observed slow growth in the media. The insignificant differences between performances of COMBO and BBM may however be linked to the presence of vanadium and selenium, which are well-known growth stimulants [46]. However, based on the element composition of the low-cost COMBO media, the growth of the algae may be limited when the culture improves to high cell concentrations. This is a possible risk that needs to be tested in future research.

**Figure 2:**
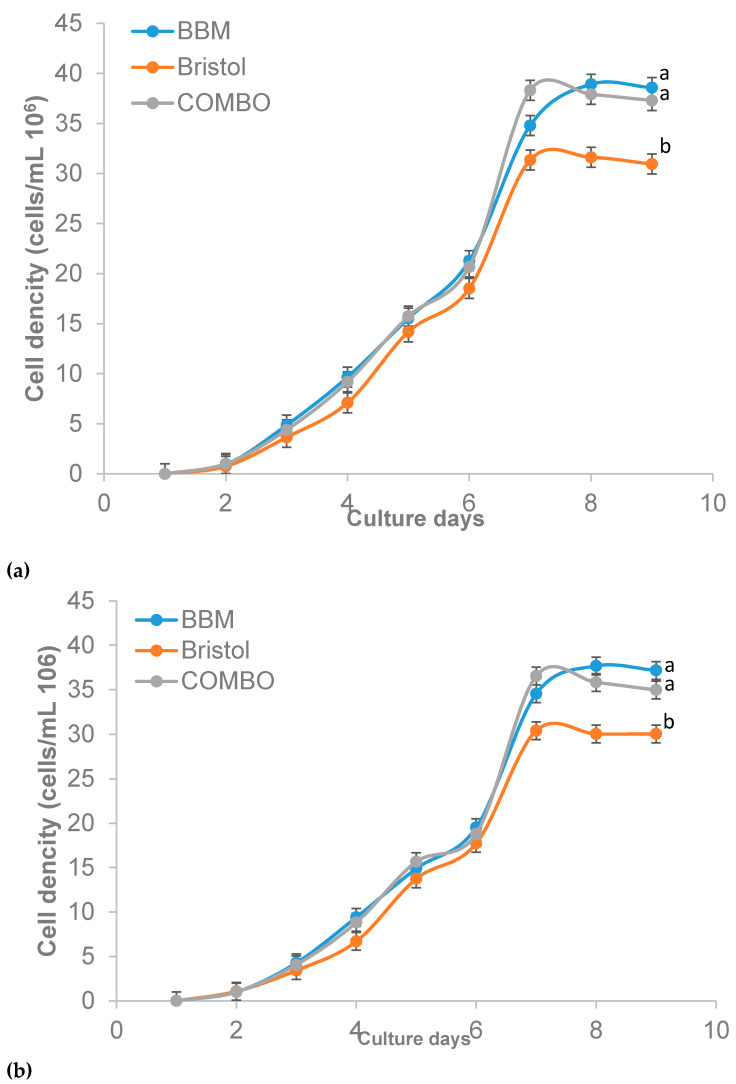
Cell density of *Ankistrodesmus falcatus* in 100% (**a**) and 50% (**b**) concentration (means ± standard errors). Line with different lowercase letters differs significantly (*p* ≤ 0.05).

**Figure 3:**
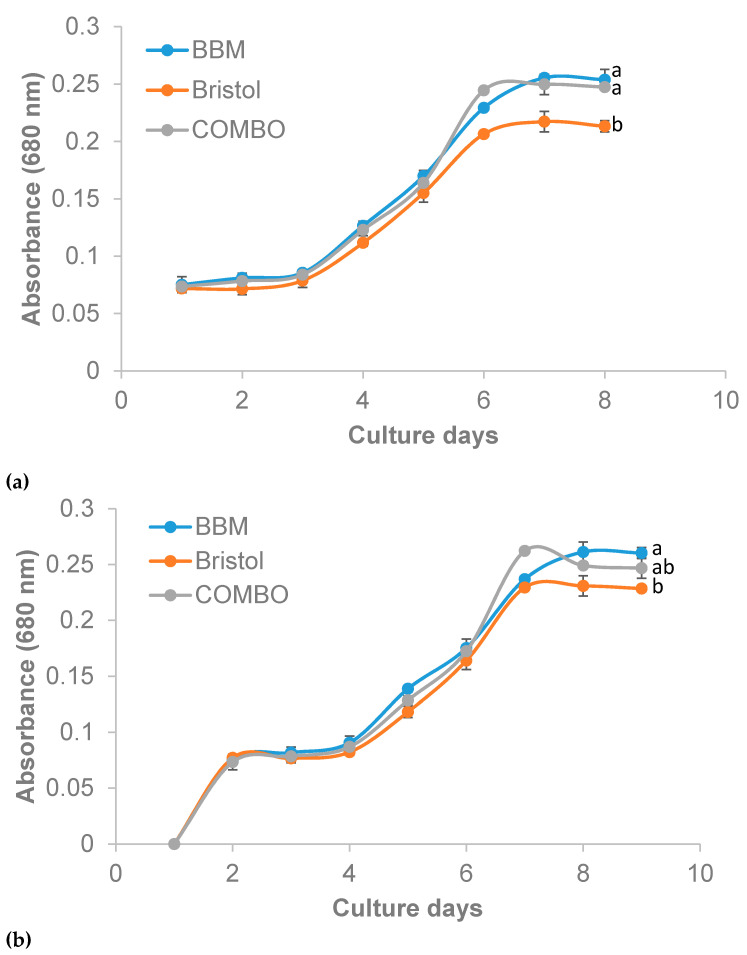
Optical density of *Ankistrodesmus falcatus* in 100% (**a**) and 50% (**b**) concentration (means ± standard errors). Line with different lowercase letters differs significantly (*p* ≤ 0.05).

**Figure 4:**
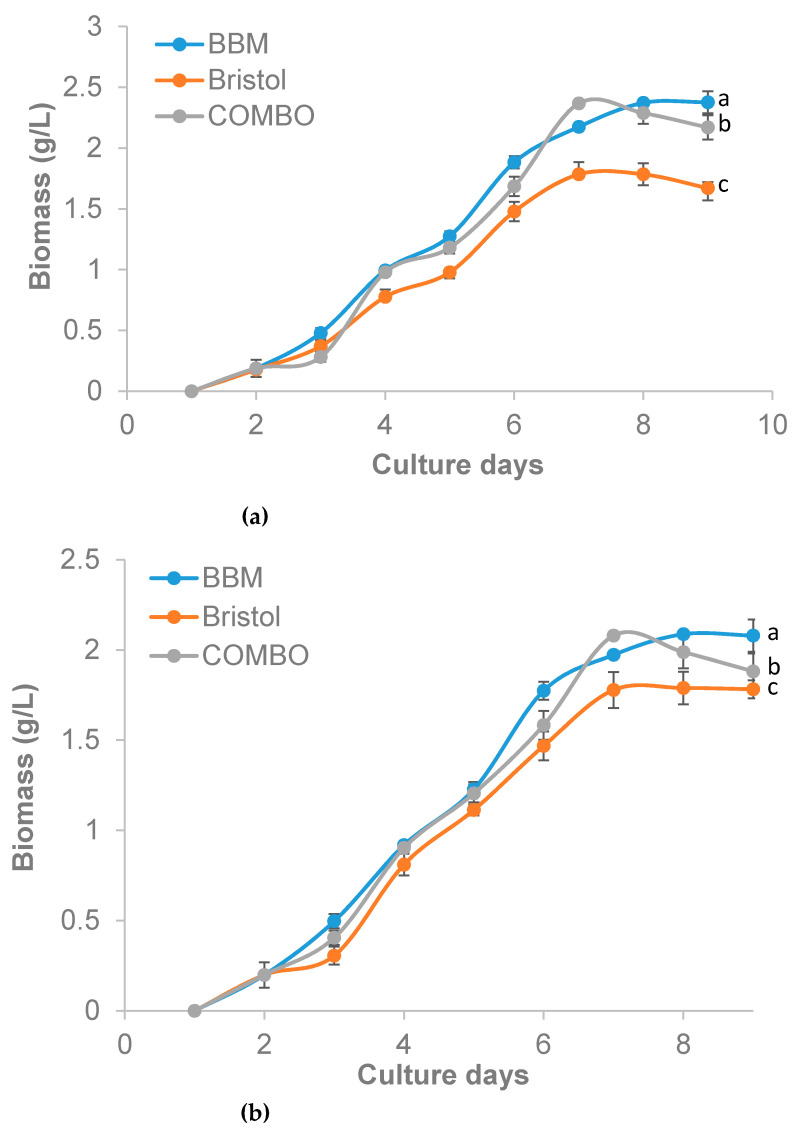
Biomass of *Ankistrodesmus falcatus* in 100% (**a**) and 50% (**b**) concentration (means ± standard errors). Line with different lowercase letters differs significantly (*p* ≤ 0.05).

Generally, the growth of microalgae goes through four different phases, namely, the lag phase, exponential phase, stationary phase, and lysis phase [10,47]. This was evident in the current study with growth exponentially increasing until the seventh (COMBO and Bristol) and eighth days (BBM) (Figure 2, Figure 3 and Figure 4). In the study by Sipauba-Tavares and Pereira [48], *Ankistrodesmus gracilis* was reported to have grown exponentially until the sixth day with a harvest of 1.44 × 10^6^ cells/mL through indoor culture using NPK (Nitrogen; Phosphorus and Potassium) medium. However, after the sixth day, the algal number decreased to 9.0 × 10^5^ cells/mL. The differences in the performances observed can be linked to differences in the microalgae species and preferences for growth media used, as demonstrated in our study. Grimm and Fisher [49] had earlier opined that growth limitation would be observed in benthic algae (including *Ankistrodesmus* sp.) if the concentration of nutrients is reduced. The finding of the current study is however suggestive that *A. falcatus* can flourish ideally with a 50% limitation of BBM, Bristol, and COMBO. This finding is very important in reducing the cost of microalgae production.

### 3.2. Effect of Culture Media on the Proximate Composition of A. falcatus

Among the different algal properties used to estimate the physiological states of phytoplankton, the biochemical composition is more of a useful physiological indicator even when species-specific variability is considered [50]. Biochemical studies include the proximate composition, among other factors [51]. Several strategies have been researched in an attempt to improve microalgae lipid, carbohydrate, and protein content. These include but are not limited to identifying the best culture media and composition as well as other physical parameters that affect microalgae performance, i.e., pH, photoperiod, salinity, etc. [5,52]. The present study observed that the lipid and protein content of microalgae in *A. falcatus* (Table 3) was significantly higher in BBM at 100% concentration (23%) than the other media (21%). However, there was no significant difference in all the media in the 50% concentration for these parameters (22%). For carbohydrates, BBM had the highest value in both concentrations tested.

The lipid content of *A. falcatus* herein is lower than the dry weight range of 24–31% reported in some earlier studies [4,52,53,54,55]. High lipid content of 43.3% had been recorded for *A. falcatus* grown in BBM under NaCl stress conditions by Talukdar et al. [46]. George et al. [44] had also reported that *A. falcatus* yielded more than 35% in total lipids in BG-11 medium under optimum light and photoperiod conditions. Generally, the total lipid of many microalgae is between the range of 20–50% of the dry biomass weight [56]. Several studies showed that cell lipid content varies because of changes in growth conditions or nutrient concentration [57,58]. Hence, the increase in lipid content normally occurs as a response to different culture conditions [59]. This may justify the differences observed herein for the different culture media.

As a general rule of thumb, the protein content of algal is between 16% and 70% dry weight, as opined by Brown et al. [60]. The protein content of *A. gracilis* was more than 50% in total of the dry weight in NPK [61]. However, in our study, the protein content of *A. falcatus* grown in all the media ranged between 43% and 46%. In a pyramid lake medium culture, the findings of Tornabene [62] showed that *A. falcatus* had a lower protein content of 31.1%. Habib et al. [63] on the other hand observed a range of 38–43.5% for *A. convolutus* cultured in different concentrations of rubber media sorts. Under control condition, *A. gracilis* produced a 47% protein in a CHU_12_ medium [61], while protein content of *A. falcatus* increased (52%) under stress conditions of NaCl [64]. In an opposing trend, the carbohydrate content of *A. falcatus* tends to reduce (14.5% and 13.5%, respectively) under stress conditions (i.e., salinity) using freshwater medium BG-11 and BBM [46]. In the present research, the carbohydrate production of *A. falcatus* was between 28% and 32% in both 50% and 100% treatments. The biomass composition of microalgae has been reported to vary with different medium compositions and under different culture conditions [65], hence the reason for the observed differences herein.

### 3.3. Effect of Culture Media on Microelement Concentration of A. falcatus

The mineral composition is among many important parameters that determine the economic feasibility of any microalgae species for its alternative use [24]. Major nutrients such as Mg^2+^, Ca^2+^, K^+^, and P^5+^ are used by microalgae as a component of the cell, while the minor nutrients such as Fe^3+^, Zn^2+^, Mn^4+^, and Cu^2+^ are essential in microalgae cells as either enzyme co-factors or as a component for its chlorophyll formation [66]. Thus, when nutrient contents of the growth media are compared, the amount of each element in the microalgae observed (see Table 4) seem to conform to the various addition or deficit of macro- and micro-nutrients in the different growth media. The deficiency of iron and magnesium in Bristol media for instance may not have only resulted in the lower accumulation of these minerals, but also may have affected growth due to the effect of these minerals on photosynthetic rate [67]. This is because iron acts as the redox catalyst in photosynthesis and nitrogen assimilation, thereby participating in the electron transport reactions of the photosynthetic organisms [68]. Magnesium is also an essential component of chlorophyll; hence, it is instrumental for the formation of catalase in microalgae. The limitation of these minerals will therefore interrupt their accumulation, as well as general cell division and growth of the algae [69]. There is a paucity of information on the elemental composition of *Ankistrodesmus* sp., however, our study (Table 4) compares favorably with those reported by International Atomic Energy Agency [70] on major and minor elements naturally occurring in algae. The minor differences may be linked to the indoor laboratory conditions (such as CO_2_ resource, light intensity, and nutrients) under which the current study was done.

## 4. Conclusions

Considering the finding obtained for growth and nutritional composition in the current study, it is concluded that BBM and COMBO are better media for the culture of *A. falcatus*. This is because cell density, optical density, specific growth rate, protein, lipid, and several mineral contents of the *A. falcatus* raised in BBM and COMBO were significantly higher than those cultured in Bristol. It is also interesting to note that the performance in both concentrations used was similar, hence suggesting that half the culture concentration can be used to propagate the *A. falcatus,* thereby reducing production cost. However, it is important to state that experimental mass production of the microalgae using a 50% concentration of the medium would be necessary to validate this finding. Also, the implication of feeding aquaculture species (both fin fish and shellfish) with the microalgae grown on different media (either directly as starter feed or included as a component of the diet) can be the focus of future research.

## Figures and Tables

**Table 1:** Media preparation of Bold’s Basal Medium (BBM) [32], Bristol [33], and Modified COMBO Medium (COMBO) [34].

Reagents	Stock Solution g/L			Quantity mL/L		
	BBM	Bristol	COMBO	BBM	Bristol	COMBO
KH_2_PO_4_	17.5	17.5	-	10.0	10.0	-
CaCl_2_·2H_2_O	2.5	2.5	36.76	10.0	10.0	1.0
MgSO_4_·7H_2_O	7.5	7.5	36.76	10.0	10.0	1.0
NaNO_3_	25.0	25.0	85.01	10.0	10.0	1.0
K_2_HPO_4_	7.5	7.5	8.71	10.0	10.0	1.0
NaCl	2.5	2.5	-	10.0	10.0	-
Na_2_SiO_3_·9H_2_O	-	-	2.842	-	-	1.0
NaHCO_3_	-	-	12.60	-	-	1.0
KCL	-	-	7.45	-	-	1.0
Na_2_EDTA·2H_2_O	10.0	-	-	1.0	-	-
KOH	6.2	-	-	1.0	-	-
FeSO_4_·7H_2_O	4.98	-	-	1.0	-	-
H_2_SO_4_ (conc.)	1 mL/L	-	-	1.0	-	-
H_3_BO_3_	11.5	-	1.0	0.7	-	1.0
**Trace Metal Solution**						
H_3_BO_3_	-	-	1.0	-	-	1.0
MnC_l2_·4H_2_O ZnSO_4_·7H_2_O	2.86 -	- -	- 1.0	1.0 -	- -	- 1.0
Na_2_MoO_4_·2H_2_O CuSO_4_·5H_2_O Co(NO_3_)_2_·6H_2_O	1.81 - -	- - -	180.0 1.0 1.0	1.0 - -	- - -	1.0 1.0 1.0
CoCl_2_·6H_2_O Na_3_VO_4_	0.222 -	- -	22.0 1.0	1.0 -	- -	1.0 1.0
H_2_SeO_3_ Na_2_EDTA·2H_2_0	0.390 -	- -	6.0 0.5	1.0 -	- -	1.0 1.0
FeCl_3_	0.079	-	10.0	1.0	-	1.0
Vitamin Solution: Thiamine·HCl (Vit. B_1_)	- -	- -	1.0 1.0	- -	- -	1.0 1.0
Biotin (Vit. H)	-	-	0.5	-	-	1.0
Cyanocobalamin (Vit. B_12_)	-	-	0.55	-	-	1.0

**Table 2:** Mean growth parameters of *Ankistrodesmus falcatus* cultured in different media. Numbers are means ± standard errors. ^a–c^ Value under the same parameter represents the mean samples with a significant difference between culture media (*p* ≤ 0.05). * Value under the same parameter represents the mean samples with a significant difference between concentrations (*p* ≤ 0.05).

Parameter	Media Concentration	
	100%	50%
**Cell Density; Cells/mL (×10^6^)**		
BBM	39.00 ± 0.58 ^a^	37.00 ± 0.58 ^a^
COMBO	37.67 ± 1.45 ^a^	35.00 ± 1.73 ^a^
Bristol	31.33 ± 0.88 ^b^	30.00 ± 0.58 ^b^
**Optical Density (680 nm)**		
BBM	0.26 ± 0.002 ^a^	0.26 ± 0.002 ^a^
COMBO	0.25 ± 0.017 ^a^	0.24 ± 0.008 ^ab^
Bristol	0.22 ± 0.004 ^b^	0.23 ± 0.002 ^b^
**Biomass Dry Weight (g/L)**		
BBM	2.33 ± 0.06 ^a^	2.20 ± 0.01 ^a^
COMBO	2.13 ± 0.06 ^b,^*	1.87 ± 0.06 ^b,^*
Bristol	1.73 ± 0.06 ^c^	1.70 ± 0.01 ^c^
**Specific Growth Rate (%/day)**		
BBM	0.443 ± 0.001 ^a^	0.437 ± 0.002 ^a^
COMBO	0.439 ± 0.003 ^a^	0.429 ± 0.002 ^a^
Bristol	0.416 ± 0.004 ^b^	0.411 ± 0.006 ^b^

**Table 3:** Proximate composition of *Ankistrodesmus falcatus* cultured in different media. Numbers are means ± standard errors. ^a–c^ Value under the same parameter represents the mean samples with a significant difference between culture media (*p* ≤ 0.05). * Value under the same parameter represents the mean samples with a significant difference between concentrations (*p* ≤ 0.05).

Parameter	Media Concentration	
	100%	50%
**Protein (%)**		
BBM	46.41 ± 0.57 ^a,^*	44.25 ± 0.26 ^a,^*
COMBO	45.99 ± 0.15 ^a,b,^*	43.45 ± 0.26 ^a,^*
Bristol	44.37 ± 0.65 ^b^	43.48 ± 0.51 ^a^
**Lipid (%)**		
BBM	23.22 ± 0.56 ^a^	22.93 ± 0.58 ^a^
COMBO	21.70 ± 0.63 ^b^	22.29 ± 0.57 ^a^
Bristol	21.64 ± 0.21 ^b^	21.28 ± 0.52 ^a^
**Carbohydrate (%)**		
BBM	32.99 ± 0.26 ^a^	32.11 ± 0.07 ^a^
COMBO	31.23 ± 0.05 ^b^	30.79 ± 0.02 ^b^
Bristol	29.76 ± 0.62 ^b^	28.93 ± 0.24 ^c^

**Table 4:** Mineral composition of *Ankistrodesmus falcatus* cultured in different media. Numbers are means ± standard errors. ^a–c^ Value under the same parameter represents the mean samples with a significant difference between culture media (*p* ≤ 0.05). * Value under the same parameter represents the mean samples with a significant difference between concentrations (*p* ≤ 0.05).

Element (mg/L)	Media Concentration		Element (mg/L)	Media Concentration	
	100%	50%		100%	50%
**Mg^+2^**			**Na^+^**		
BBM	555.76 ± 8.85 ^a,^*	477.14 ± 2.94 ^a,^*	BBM	364.54 ± 1.27 ^a,^*	132.21 ± 2.45 ^a,^*
COMBO	301.57 ± 7.34 ^b,^*	431.56 ± 7.80 ^b,^*	COMBO	281.76 ± 4.53 ^b,^*	131.75 ± 1.25 ^a,^*
Bristol	279.16 ± 1.72 ^b^	205.95 ± 6.35 ^c^	Bristol	133.20 ± 0.86 ^c,^*	80.29 ± 1.85 ^b,^*
**Cr^+3^**			**Al^+3^**		
BBM	4.91 ± 0.18 ^a^	4.12 ± 0.07 ^a^	BBM	92.73 ± 0.71 ^a,^*	47.22 ± 1.58 ^a,^*
COMBO	2.49 ± 0.21 ^b,^*	3.76 ± 0.14 ^a,^*	COMBO	15.28 ± 0.09 ^b,^*	32.84 ± 0.96 ^b,^*
Bristol	3.20 ± 0.09 ^b^	3.13 ± 0.04 ^b^	Bristol	11.01 ± 0.07 ^c,^*	23.18 ± 0.63 ^c,^*
**Fe^+3^**			**Mn^+4^**		
BBM	235.86 ± 1.27 ^a,^*	124.66 ± 1.64 ^a,^*	BBM	29.08 ± 0.54 ^a,^*	21.87 ± 0.44 ^a,^*
COMBO	32.64 ± 0.23 ^c,^*	91.27 ± 1.60 ^b,^*	COMBO	2.97 ± 0.24 ^c,^*	13.85 ± 0.07 ^b,^*
Bristol	81.06 ± 1.22 ^b,^*	50.25 ± 0.35 ^c,^*	Bristol	6.33 ± 0.14 ^b^	4.16 ± 0.09 ^c^
**Zn^+2^**			**Co^+2^**		
BBM	7.01 ± 0.26 ^a,^*	4.94 ± 0.34 ^a,^*	BBM	0.28 ± 0.010 ^a,^*	0.12 ± 0.008 ^a,^*
COMBO	2.68 ± 0.04 ^b,^*	5.22 ± 0.25 ^a,^*	COMBO	0.03 ± 0.001 ^b,^*	0.07 ± 0.005 ^b,^*
Bristol	3.69 ± 0.17 ^b^	3.58 ± 0.37 ^a^	Bristol	0.09 ± 0.001 ^b^	0.06 ± 0.006 ^b^
**Se^+2^**			**Cu^+2^**		
BBM	0.21 ± 0.001 ^a,^*	0.06 ± 0.001 ^b,^*	BBM	3.64 ± 0.16 ^a^	3.39 ± 0.04 ^a^
COMBO	0.09 ± 0.001 ^b^	0.06 ± 0.001 ^b^	COMBO	0.48 ± 0.03 ^c,^*	1.83 ± 0.03 ^b,^*
Bristol	0.07 ± 0.001 ^c^	0.08 ± 0.002 ^a^	Bristol	1.22 ± 0.004 ^b,^*	1.04 ± 0.008 ^c,^*
**Pb^+2^**			**Cd^+2^**		
BBM	0.25 ± 0.002 ^a,^*	0.34 ± 0.001 ^a,^*	BBM	0.01 ± 0.001 ^c^	0.02 ± 0.001 ^c^
COMBO	0.06 ± 0.002 ^c,^*	0.12 ± 0.001 ^b,^*	COMBO	0.05 ± 0.002 ^a^	0.06 ± 0.005 ^a^
Bristol	0.11 ± 0.001 ^b^	0.09 ± 0.002 ^c^	Bristol	0.02 ± 0.001 ^b,^*	0.04 ± 0.006 ^a,^*

## Data Availability

The data presented in this study are available on request from the corresponding authors.
